# Intratumoral serotonin and antidepressants in glioblastoma patients: narrowing the uncertainties

**DOI:** 10.1007/s11060-026-05694-1

**Published:** 2026-06-30

**Authors:** Wendy Yi-Ying Wu, Barbro Numan Hellquist, Beatrice Melin, Benny Björkblom, Rickard L. Sjöberg

**Affiliations:** 1 Department of Diagnostics and Intervention, Oncology, Umeå University, Umeå, 90187 Sweden; 2 Department of Chemistry, Umeå University, Umeå, 90187 Sweden; 3 Department of Clinical Science, Neurosciences, Umeå University, Umeå, 90187 Sweden

**Keywords:** Glioblastoma, Tissue metabolites, Antidepressant use, Survival

## Abstract

**Purpose:**

Antidepressant use, which targets intracerebral serotonin metabolism, is common among glioblastoma patients. However, its interaction with tumor metabolism and survival remains unclear. We investigated the relationships between antidepressant use, intratumoral serotonin pathway metabolites, quality-of-life, and survival.

**Methods:**

We analysed two complementary cohorts: a population-based cohort (*n* = 801) and a hospital-based biobank cohort (*n* = 153). In the population-based cohort, information about antidepressant use and survival were obtained from national registers. In the hospital-based cohort, intratumoral serotonin pathway metabolites were measured, and data on preoperative antidepressant use and quality-of-life were recorded. Linear and Cox regression models were used to investigate the association between antidepressant use, metabolites, quality-of-life and survival.

**Results:**

In the population-based cohort, antidepressant use was associated with poorer survival in adjusted analyses. However, use of fluoxetine or sertraline was associated with better survival compared with other selective serotonin reuptake inhibitors (HR = 0.62, 95% CI = 0.44–0.88). In the hospital-based cohort, preoperative antidepressant use was associated with lower intratumoral serotonin levels and its downstream metabolite, 5-HIAA. Higher serotonin levels were associated with better preoperative quality-of-life, especially general health. Serotonin pathway metabolites were not clearly associated with survival.

**Conclusions:**

These findings suggest that higher tumor tissue serotonin abundance was associated with better patient-reported well-being but not with survival in gliobastoma. Survival differed across SSRI exposure groups, although causal interpretation is limited by the observational design. These findings do not provide strong evidence against the continued clinical use of sertraline or fluoxetine in glioblastoma patients when indicated.

**Supplementary Information:**

The online version contains supplementary material available at 10.1007/s11060-026-05694-1.

## Introduction

Glioblastoma is a relatively common, diffuse growing, highly malignant primary brain tumor with a five-year survival of approximately 5% even after surgery and radiochemotherapy [1]. Use of antidepressant treatment is more common amongst glioma patients than in the general population [2]. Both gliomas, antidepressants and depression/anxiety themselves are known to interact with neural transmission in ways that are still not fully understood [3–6]. Despite its high clinical relevance, the question whether antidepressant treatment may also interact with tumor metabolism to affect survival in glioma remains unanswered [7–10]. Both beneficial and harmful pharmacological effects of these medications have been proposed.

Support for beneficial effects of certain antidepressants comes primarily from preclinical studies, which have focused on mechanisms beyond serotonergic transmission. In one such study, Bi et al. demonstrated that fluoxetine, a selective serotonin reuptake inhibitor (SSRI), inhibits sphingomyelin phosphodiesterase 1 (SMPD1) leading to inhibition of epidermal growth factor signaling, thereby killing glioma cells in a mouse model [11]. In the same study, survival was also examined in a small clinical cohort, with longer survival observed among 10 glioblastoma patients treated with fluoxetine compared with 182 untreated patients. Sánchez-Castillo et al. found that sertraline, another commonly prescribed SSRI, selectively impairs nucleotide synthesis and energy production in subsets of glioblastoma cells that depend on the Serine/Glycine pathway for energy production [12].

On the other hand, poorer survival associated with non-SSRI agents, particularly in glioblastoma, were recently found in one systematic review and meta-analysis [7]. Similarly, a subsequent meta-analysis focusing exclusively on glioblastoma reported no significant association between antidepressant use and overall survival [10]. Observational cohorts have reported divergent findings, from improved survival with specific SSRI, such as fluoxetine [11], to worse outcomes associated with antidepressant use [8]. Methodological differences across studies— including study design, definition of antidepressant use, and susceptibility to immortal time bias— likely contribute to observed heterogeneity. Interestingly, following recent meta-analyses, a large single-center cohort study of 1464 consecutive glioblastoma patients reported that antidepressant use as a class was associated with worse survival in glioblastoma after adjusting for key clinical and molecular prognostic factors, while opposite trends were found for fluoxetine and sertraline in the same cohort [13].

Major classes of antidepressants are believed to exert their therapeutic effects by increasing serotonin levels in the central nervous system, thereby improving mental health and subjective quality-of-life (QoL) in patients with pathologically low levels of this neurotransmitter [14, 15]. In parallel, a growing body of evidence suggests that serotonin transmission may be involved in glioma development and progression [16–21]. Extending these findings to human tumor tissue, the first study to directly evaluate this association reported that glioma patients who used antidepressants preoperatively had significantly lower intratumoral serotonin levels than patients not receiving antidepressant treatment [6]. Because intratumoral serotonin levels prior to antidepressant initiation were not available, it remains unclear whether antidepressant therapy reduced tumor serotonin levels relative to baseline, which would be pharmacologically unexpected. The observed association may instead reflect confounding by indication, whereby underlying differences in QoL or symptom burden influences both serotonergic biology and the likelihood of antidepressant use. While that study demonstrated an association between antidepressant use and intratumoral serotonin levels, it did not evaluate patient-reported QoL, association between serotonin pathway metabolites and survival. These limitations underscore the need to simultaneously evaluate QoL, serotonin levels, antidepressant use, and survival outcomes in glioblastoma patients. To address these unsolved issues, the present study was designed to clarify the interrelationship between serotonergic biology, antidepressant exposure, and clinical outcomes in glioblastoma. First, we investigated whether use of the SSRIs fluoxetine or sertraline was associated with improved survival compared with other SSRIs in a population-based cohort, accounting for time-varying antidepressant use. Second, we examined the association between preoperative antidepressant use and intratumoral concentration of metabolites in the serotonin pathway, including tryptophan, serotonin, 5-hydroxyindoleacetic acid (5-HIAA), kynurenine, and the 5-HIAA/serotonin ratio as a marker of serotonin turnover. The association between intratumoral metabolites and overall survival was further investigated. Third, we assessed whether intratumoral serotonin pathway metabolites were associated with preoperative QoL, thereby evaluating potential confounding by symptom burden.

## Methods

### Data source

This study is based on two complementary data sources: (1) a population-based cohort (RISK North) derived from Swedish national health registers, and (2) a hospital-based biobank of glioblastoma patients with available tumor tissue.

The population-based cohort was obtained from the RISK North database, which integrates information from several Swedish national registers. This cohort includes 801 patients diagnosed with grade 4 glioma between 2009 and 2013 according to the diagnostic criteria used during this study period. The register-based data provides detailed information on diagnoses, treatments, prescription drug use, and survival, enabling longitudinal follow-up at the population level [22]. Information about pharmacy dispensations of antidepressant was obtained from the Swedish Prescribed Drug register and served as a proxy for antidepressant use and information about overall survival was obtained from the Swedish cause of death register. The glioma patients were followed from date of diagnosis (2009–2013) until death or end of follow-up (2019-12-31).

The hospital-based cohort comprises 153 patients with glioblastoma diagnosed between 2004 and 2016, from whom tumor tissue was obtained during neurosurgical procedures at Umeå University Hospital. Glioma classification was performed according to the 2016 WHO classification of Tumors of the Central Nervous System and subsequent cIMPACT-NOW recommendations implemented in the WHO 2021 classification. Tumor tissue was collected during surgery and frozen within 30–60 min and stored at − 80 °C until further analysis. Information on WHO performance status and current medication use was recorded 1–4 days prior to the first surgery for all patients as part of the admission routine and survival status was ascertained on August 15, 2024, through electronic medical records linked to the Swedish national population registry. IDH mutation status and MGMT promoter methylation status were obtained from previously performed molecular analyses, as described in detail elsewhere [23]. Briefly, IDH status was determined using multiplex ligation-dependent probe amplification targeting common IDH1 and IDH2 mutations. MGMT promoter methylation was assessed by pyrosequencing and categorized as unmethylated (< 10%), low methylation (10–25%), or high methylation (> 25%) according to established criteria [24]. Four metabolites involved in tryptophan metabolism— tryptophan, serotonin, 5-HIAA, and kynurenine— were included in the analyses. Additionally, the 5-HIAA/serotonin ratio was calculated to explore potential alterations in serotonin metabolism. These metabolites were measured as part of a previously published metabolomics dataset using gas chromatography-mass spectrometry (GC-MS) and liquid chromatography-mass spectrometry (LC-MS) with full methodological details reported elsewhere [23]. In brief, samples were randomized prior to analysis according to key clinical and technical variables, and pooled quality control samples were analyzed repeatedly throughout the analytical runs to monitor analytical performance. Metabolite peak intensities were normalized using internal standards, accounting for potential variation introduced by sample handling, batch differences, or instrument fluctuations. Missing values attributed to biological factors or measurements below the detection limit were imputed using half the lowest observed value.

For a subset of this cohort (*n* = 46), preoperative QoL data was available through a routine that was gradually implemented between 2005 and 2010 (See Fig S1). QoL was assessed using the European Organization for the Research and Treatment of Cancer Core Quality-of-Life Questionnaire (EORTC QLQ-C30). For the present study, we focused on item 29 (How would you rate your overall health during the past week?) and 30 (How would you rate your overall quality-of-life during the past week?). Answers to these questions are recorded on a 7-point rating scale and analyses incorporated each item separately as well as the mean of the two items as a summary QoL measure.

### Statistical analysis

To investigate the association between postoperative antidepressant exposure and overall survival, we accounted for the time-varying nature of antidepressant use to avoid immortal time bias. Patients were considered unexposed until their first recorded dispensation after diagnosis and exposed thereafter. Antidepressant use in the 6 months preceding diagnosis was included as a baseline covariate to account for prior treatment. Patients contributed person-time to exposure states defined by antidepressant use over follow-up; exposure was categorized as fluoxetine or sertraline (ATC code, N06AB03 or N06AB06), other SSRIs (ATC code, N06AB04, N06AB05, and N06AB10) or no SSRI use. The time-dependent Cox proportional hazards regression was used to estimate hazard ratios while adjusting for age at diagnosis, sex, type of surgery and WHO performance status. Adjusted survival curves represent the predicted survival under fixed exposure categories, based on hazard estimates from the time-dependent Cox proportional hazards model to illustrate survival patterns. To evaluate the potential influence of reverse causation and antidepressant prescribing near the end of life, sensitivity analyses were performed using 30-day and 90-day lag periods. In these analyses, antidepressant exposure was shifted forward by 30 or 90 days, respectively, such that prescriptions dispensed within the lag period were not considered to contribute to the exposed risk period.

Distribution of log2-transformed metabolites, including tryptophan, serotonin, 5-HIAA, kynurenine and ratio of 5-HIAA/serotonin, were summarized by antidepressant use using box plots and compared with the Wilcoxon rank-sum test. Each metabolite was analyzed in a separate multivariable linear regression comparing antidepressant users with non-users. Linear regression was used to evaluate the association between preoperative antidepressant use and intratumoral metabolite levels. Models were adjusted for age at operation, sex, and IDH status, as IDH mutations are known to influence metabolic pathways in glioma. Global QoL was derived from EORTC QLQ-C30 as the means of items Q29 and Q30. Associations between metabolites and Global QoL were evaluated using multivariable linear regression, while Q29 and Q30 were additionally analyzed as ordinal outcomes using cumulative link models. Adjusted analyses included age at operation and sex (Model 1), with a second exploratory model additionally adjusted for WHO performance status and IDH status (Model 2). Cox proportional hazards models were used to examine associations between metabolites and overall survival. Adjusted analyses included age at operation, sex, and type of surgery (Model 1), with a second model additionally adjusted for IDH status, MGMT promoter methylation status, and WHO performance status (Model 2). Analyses were performed using R version 4.5.2.

## Results

### Baseline characteristics

Table 1 showed the baseline characteristics for two cohorts. The population-based cohort included 801 glioblastoma patients diagnosed in Sweden between 2009 and 2013. A total of 153 patients were included in the hospital-based cohort, of whom 46 comprised the sub-cohort with available preoperative QoL data. In the hospital-based cohort, the median age at diagnosis was 62 years (Q1–Q3: 52–71), similar to the QoL sub-cohort (60 years, Q1–Q3: 50–67) and the population-based cohort (64 years, Q1–Q3: 56–71). Males constituted 65% of the hospital-based cohort and 78% of the QoL sub-cohort, whereas 61% of patients in the population-based cohort were male. In the population-based cohort, 88% of patients had a WHO performance status of 0–2. In the hospital-based cohort, 94% of patients had IDH-wildtype tumors, 67% had unmethylated MGMT promoter status, and 95% had a WHO performance status of 0–2.

**Table 1:** Baseline characteristics of patients in the hospital-based and population-based glioblastoma cohorts

Characteristic	Population-based cohort	Hospital-based cohort	
	(*n* = 801)	Whole (*n* = 153)	Sub-cohort with QoL† (*n* = 46)
Age, median (Q1, Q3)*	64 (56, 71)	62 (52, 71)	60 (50, 67)
Male sex, n (%)	491 (61%)	99 (65%)	36 (78%)
Calendar year, n (%)*			
2004–2006	-	40 (26.1%)	1 (2.2%)
2007–2009	147 (18.4%)	52 (34.0%)	4 (8.7%)
2010–2012	496 (61.9%)	27 (17.6%)	16 (34.8%)
2013–2016	158 (19.7%)	34 (22.2%)	25 (54.3%)
Type of surgery, n (%)		-	-
Resection	598 (74.7%)	132 (86.3%)	40 (87%)
Biopsy	203 (25.3%)	21 (13.7%)	6 (13%)
IDH status, n (%)			
Mutated	-	9 (5.9%)	4 (8.7%)
Wildtype	-	144 (94.1%)	42 (91.3%)
MGMT promoter methylation			
Un-methylated	-	103 (67.3%)	31 (67.4%)
Low-methylated	-	20 (13.1%)	8 (17.4%)
High-methylated	-	30 (19.6%)	7 (15.2%)
WHO performance status			
0–2	681 (88.1%)	146 (95.4%)	44 (95.7%)
3–4	92 (11.9%)	7 (4.6%)	2 (4.3%)
Missing	27	-	-

* age and year refer to age at operation and year of operation in the hospital-based cohort and age at diagnosis and year at diagnosis in the population-based cohort

† sub-cohort nested within the hospital-based cohort

QoL, Quality-of-life

### Associations between antidepressant use and survival

**Fig. 1:**
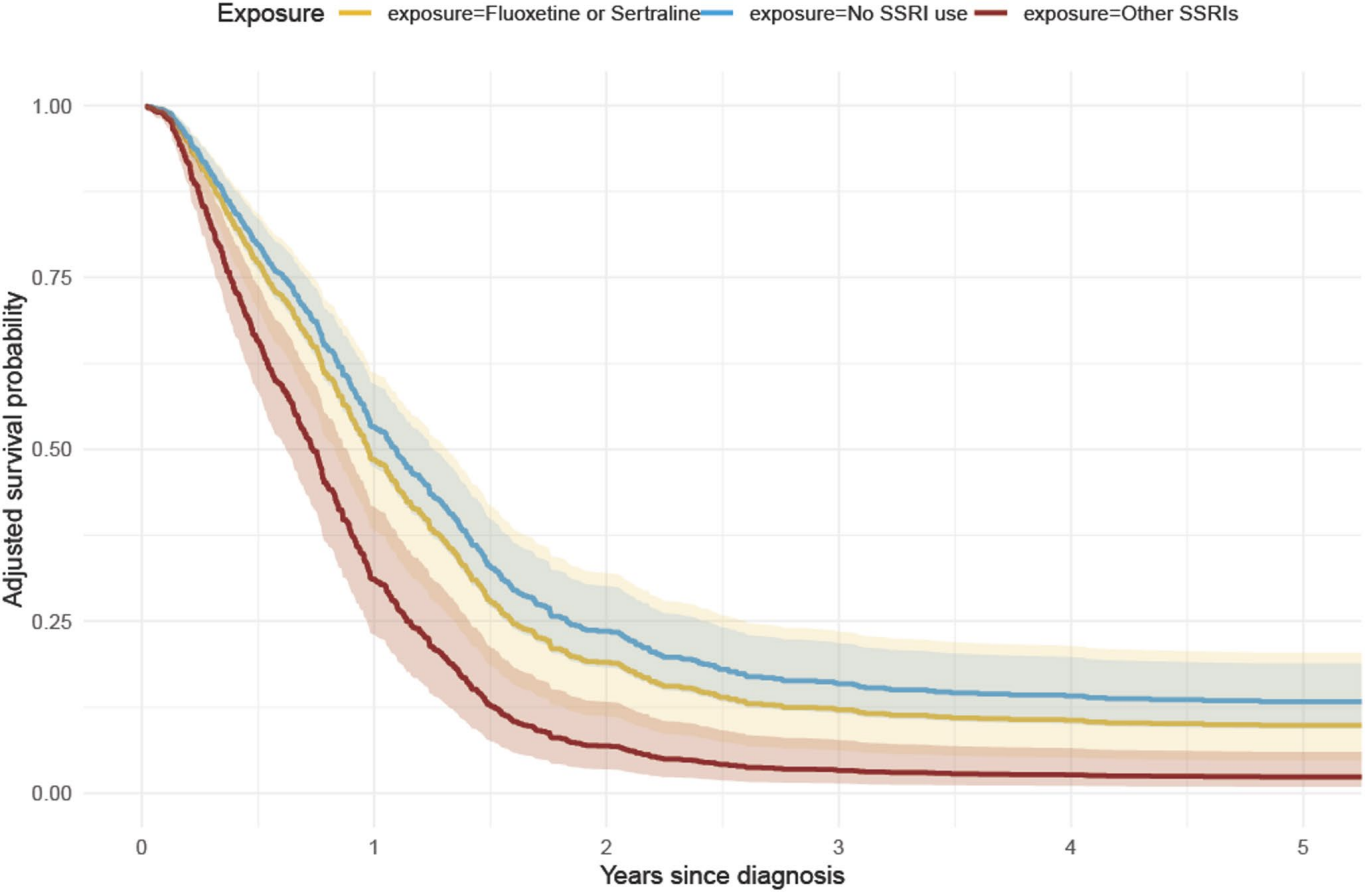
Adjusted model-based overall survival curves according to antidepressant exposure (fluoxetine or sertraline, other SSRIs and no SSRI use) Curves were predicted from the fitted Cox proportional hazards model with exposure modelled as a time-dependent covariate. Shaded areas show 95% confidence intervals

Among the 801 patients, from the population-based cohort, 60 were at some point during follow-up exposed to fluoxetine (*n* = 10) or sertraline (*n* = 50) and 123 to other SSRIs, including citalopram (*n* = 97), paroxetine (*n* = 5), escitalopram (*n* = 18), and a combination of escitalopram and citalopram (*n* = 3). Of the 60 patients exposed to fluoxetine or sertraline, 22 (37%) had received treatment during the 6 months preceding diagnosis, whereas 38 (63%) initiated treatment after diagnosis. Corresponding numbers for other SSRIs were 46 (37%) and 77 (63%). In the model including antidepressant exposure as a time-dependent variable and adjusted for age, sex, and type of surgery, exposure to fluoxetine or sertraline was associated with a lower hazard of death compared to patients exposed to other SSRIs (HR = 0.62, 95% CI = 0.44–0.88), but a higher hazard compared with no antidepressant use (HR = 1.15, 95% CI = 0.85–1.56), with the confidence interval including 1. Figure 1 showed the corresponding survival probabilities for each exposure group. In sensitivity analyses applying 30-day and 90-day lag periods, the association between fluoxetine/sertraline use and lower mortality compared with other SSRIs was attenuated but remained similar in direction (30-day lag: HR = 0.64, 95% CI = 0.45–0.91; 90-day lag: HR = 0.74, 95% CI = 0.50–1.07). Corresponding HRs comparing fluoxetine/sertraline use with no SSRI use were 1.21 (95% CI = 0.89–1.65) and 1.30 (95% CI = 0.95–1.78), respectively. These findings suggest that the main results were not solely driven by antidepressant prescriptions initiated immediately before death.

### Associations between preoperative antidepressant use and intratumoral serotonin metabolites

**Fig. 2:**
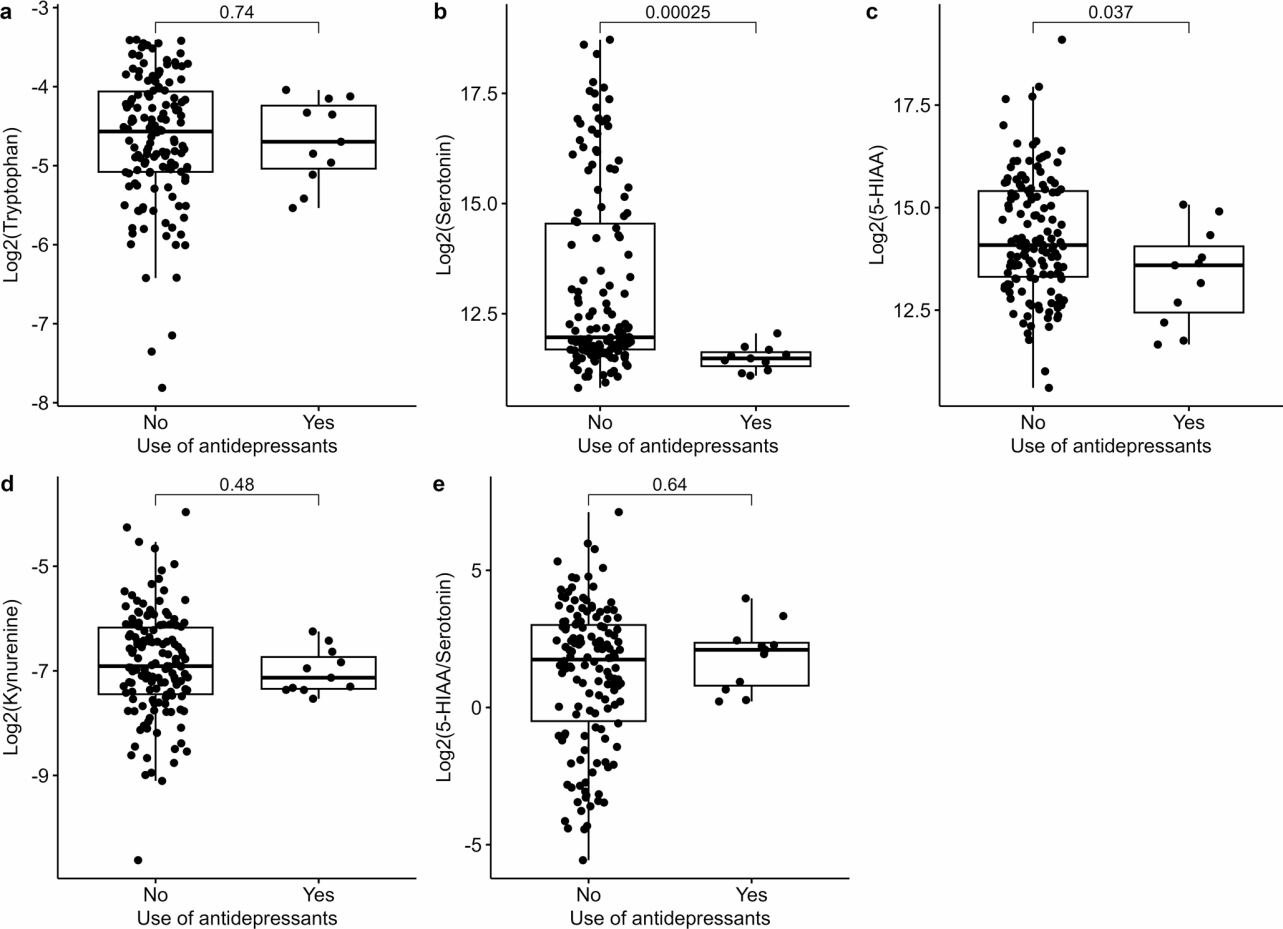
Box plot of intratumoral serotonin pathway metabolites by antidepressant use

Among 153 patients with glioblastoma in the hospital-based cohort, 11 (7.2%) reported preoperative antidepressant use. In analyses of four serotonin pathway metabolites, antidepressant use was significantly associated with lower intratumoral levels of serotonin and 5-HIAA, whereas no associations were observed for tryptophan, kynurenine, or the 5-HIAA/serotonin ratio (Fig. 2; Table 2). In the adjusted analyses, antidepressant use remained associated with lower levels of serotonin (β = −1.62, 95% CI = − 2.28 – −0.36) and 5-HIAA (β = −0.91, 95% CI = − 1.78 – −0.03).

**Table 2:** Association between antidepressant use and intratumoral metabolites

Outcome	Crude model, β (95% CI)	Adjusted model, β (95% CI)*
Tryptophan	−0.03 (− 0.53, 0.47)	−0.04 (− 0.55, 0.47)
Serotonin	−1.63 (− 2.88, − 0.38)	−1.62 (− 2.88, − 0.36)
5-HIAA	−0.95 (− 1.84, − 0.07)	−0.91 (− 1.78, − 0.03)
Kynurenine	−0.14 (− 0.75, 0.46)	−0.08 (− 0.68, 0.53)
5-HIAA/Serotonin	0.68 (− 0.88, 2.23)	0.72 (− 0.81, 2.24)

* Adjusted for age at operation, sex, an IDH status. Coefficients (β) represent the mean difference in log2-metabolite levels comparing antidepressant users with non-users

### Associations between intratumoral serotonin metabolites and survival

Table 3 shows crude and adjusted hazard ratio for overall survival in relation to each metabolite. In crude analyses, none of the investigated metabolites showed a clear association with survival. Similar results were observed after adjustment for age, sex, and type of surgery, and further adjustment for IDH status, MGMT promoter methylation status, and WHO performance status did not materially alter the estimates. Thus, no clear associations were observed between intratumoral levels of tryptophan, serotonin, 5-HIAA, kynurenine, or the 5-HIAA/serotonin ratio and overall survival.

**Table 3:** Hazard ratios for overall survival associated with intratumoral metabolites

Metabolites	Crude model, hazard ratio (95% CI)	Adjusted model 1, hazard ratio (95% CI)*	Adjusted model 2, hazard ratio (95% CI)†
Tryptophan	0.90 (0.72, 1.11)	1.02 (0.82, 1.28)	1.06 (0.85, 1.32)
Serotonin	0.97 (0.90, 1.05)	1.00 (0.92, 1.08)	0.98 (0.91, 1.07)
5-HIAA	1.00 (0.89, 1.12)	0.99 (0.88, 1.11)	1.02 (0.90, 1.15)
Kynurenine	0.94 (0.80, 1.12)	0.89 (0.74, 1.06)	0.93 (0.77, 1.12)
5-HIAA/Serotonin	1.02 (0.95, 1.08)	1.00 (0.94, 1.06)	1.02 (0.95, 1.08)

* Model 1 adjusted for age at operation, sex, and surgery type

† Model 2 adjusted for age at operation, sex, surgery type, IDH status, MGMT methylation, and WHO performance status

### Associations between intratumoral serotonin metabolites and global QoL

Table 4 summarizes crude and adjusted linear regression analyses of the association between intratumoral metabolites and the preoperative global QoL. In the crude model, higher intratumoral serotonin was associated with higher Global QoL (β = 3.11, 95% CI 0.27–5.95 per doubling of serotonin). The estimated association remained similar after adjustment for age and sex (β = 2.73, 95% CI = − 0.31–5.77) and after further adjustment for WHO performance status and IDH status (β = 3.04, 95% CI = − 0.08–6.17), although the confidence intervals included the null value. No statistically significant associations were observed for tryptophan, 5-HIAA, kynurenine, or the ratio of serotonin and 5-HIAA.

**Table 4:** Association between intratumoral metabolites and global QoL, general health and general QoL

Metabolites	Global QoL			General Health‡		General QoL‡	
	Crude model, β (95% CI)	Adjusted model 1, β (95% CI)*	Adjusted model 2, β (95% CI)†	Adjusted model 1, OR (95% CI)*	Adjusted model 2, OR (95% CI)†	Adjusted model 1, OR (95% CI)*	Adjusted model 2, OR (95% CI)†
Tryptophan	2.57 (− 6.33, 11.48)	1.58 (− 7.57, 10.74)	0.82 (− 8.29, 9.94)	0.95 (0.48, 1.89)	1.03 (0.52, 2.03)	1.12 (0.58, 2.16)	1.17 (0.61, 2.26)
Serotonin	3.11 (0.27, 5.95)	2.73 (− 0.31, 5.77)	3.04 (− 0.08, 6.17)	1.31 (1.02, 1.68)	1.38 (1.06, 1.80)	1.21 (0.95, 1.55)	1.24 (0.96, 1.60)
5-HIAA	0.09 (− 5.12, 5.30)	0.33 (− 4.90, 5.55)	−0.11 (− 5.32, 5.10)	1.15 (0.78, 1.71)	1.16 (0.78, 1.73)	0.90 (0.62, 1.30)	0.88 (0.61, 1.28)
Kynurenine	−2.25 (− 9.35, 4.85)	−1.79 (− 8.97, 5.39)	−2.98 (− 10.15, 4.18)	1.07 (0.62, 1.85)	1.04 (0.60, 1.81)	0.72 (0.43, 1.23)	0.66 (0.38, 1.15)
5-HIAA/Serotonin	−2.35 (− 4.87, 0.17)	−1.98 (− 4.66, 0.70)	−2.36 (− 5.12, 0.40)	0.85 (0.68, 1.06)	0.83 (0.66, 1.04)	0.85 (0.69, 1.04)	0.83 (0.67, 1.03)

* Model 1 adjusted for age at operation and sex

† Model 2 adjusted for age at operation, sex, WHO performance status and IDH status

**‡** ordinal regression model

OR, odds ratio

In the ordinal regression models for the general health and general QoL items, higher intratumoral serotonin was associated with higher odds of reporting better general health (OR = 1.31, 95% CI = 1.02–1.68 after adjustment for age and sex; OR = 1.38, 95% CI = 1.06–1.80 after additional adjustment for WHO performance status and IDH status) and showed a similar but less precise association with general QoL (OR = 1.24, 95% CI = 0.96 − 1.60). No statistically significant associations were observed for tryptophan, 5-HIAA, kynurenine or ratio of serotonin and 5-HIAA with either general health or general QoL.

## Discussion

In this study, we combined a population-based cohort, offering large sample size and generalizability, with a hospital-based cohort, providing detailed intratumoral metabolites measurements and preoperative QoL data, thereby enabling investigation of association across both epidemiological and molecular domains. Antidepressant SSRI use was associated with poorer survival compared with no SSRI use. However, use of fluoxetine or sertraline was associated with better survival compared with other SSRIs, while higher but no statistically significant difference was observed compared with no SSRI use. Integrating metabolomic and clinical data, we found that antidepressant use was associated with lower intratumoral serotonin levels, and higher serotonin levels were linked to better preoperative QoL. Taken together, the two cohorts provide complementary evidence regarding antidepressant exposure, serotonin-pathway metabolites, patient-reported outcomes, and survival. However, because these analyses were performed in separate cohorts, the findings should not be interpreted as demonstrating a single causal pathway.

### Effects of fluoxetine and sertraline on survival

A key finding of this study is that sertraline and fluoxetine differed from other SSRIs in being associated with significantly longer survival in glioblastoma patients. This result is consistent with previous findings reported by Sun et al. in a large clinical dataset, as well as with epidemiological data on fluoxetine reported by Bi et al. [11, 13]. However, survival among patients treated with fluoxetine or sertraline was still worse than that of patients not treated with SSRI antidepressants. This suggests that the choice of fluoxetine or sertraline as antidepressant may in part have compensated for apparent negative effects associated with SSRI use. But this was still not sufficient to have them reach the survival prospects of controls. One possible interpretation could be that use of these compounds are actually associated with beneficial effects on tumor metabolism but this potential benefit may be attenuated by confounding by indication, as antidepressants are prescribed to patients with depression or greater symptom burden —factors that may themselves be associated with poorer prognosis.

The epidemiological, registry-based nature of this dataset does not allow us to directly address whether pharmacological effects on tumor biology mediate these results. However, preclinical research has demonstrated that fluoxetine and sertraline may have antitumoral effects on glioma cell biology, including through disruptions of sphingomyelin and serine metabolism, respectively [11, 12]. Both mechanisms that have demonstrated importance in glioma oncogenesis [23, 25]. Further exploring interactions between these metabolic pathways and pharmacological substances in glioma patients is thus an important task for future studies.

Consequently, the present findings should not be interpreted as supporting initiation of fluoxetine or sertraline for antitumor purposes, nor as evidence favoring one SSRI over another in routine clinical practice. Rather, the results provide no strong evidence that continued use of fluoxetine or sertraline is harmful when these medications are clinically indicated. Decisions regarding antidepressant treatment in glioblastoma patients should remain individualized and based on established clinical considerations, including psychiatric indication, symptom control, potential drug interactions, adverse effects, concomitant medications, and overall goals of care.

### Intratumoral serotonin, QoL and survival

Another key finding of the present study was that use of antidepressants at the time of glioma surgery was significantly associated with low intratumoral serotonin levels, whereas high levels were associated with better preoperative self-reported global QoL. These results are consistent with the notion of a link between subjective well-being and intracerebral serotonin levels that have dominated thinking on mood disorders for decades [15, 26–28]. At the same time, the observed inverse association between antidepressants and serotonin levels —contrary to what might be expected based on their pharmacological effect —may reflect confounding by indication. That is, patients with a greater symptom burden or reduced well-being may be more likely both to receive antidepressants and to have lower intratumoral serotonin levels as a neural correlate of their emotional state.

**Fig. 3:**
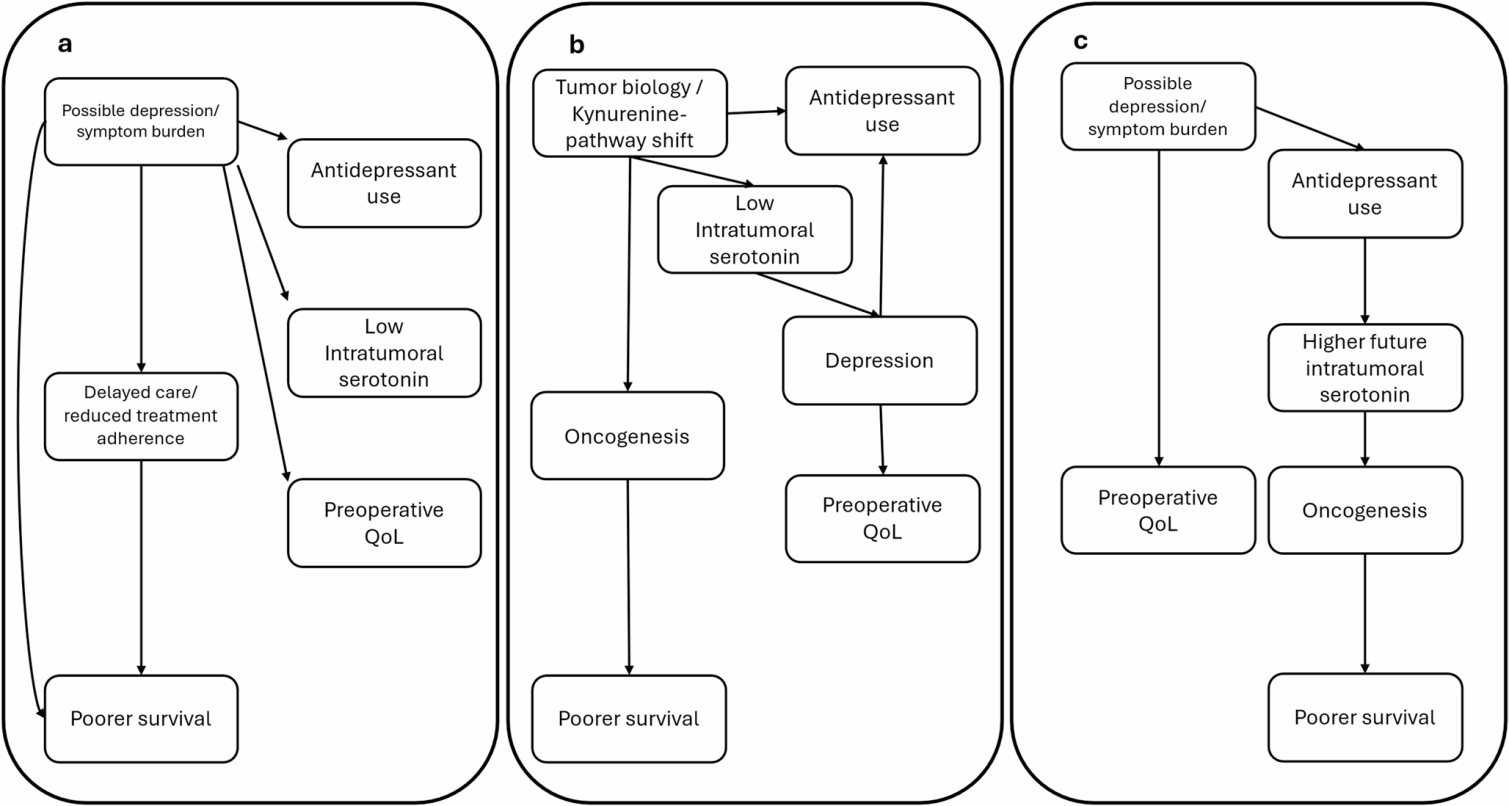
Schematic illustration of three alternative explanatory frameworks for the observed association between antidepressant use, intratumor serotonin levels, quality of life, and survival in glioblastoma patients. (**a**) a depression-related symptom burden model, (**b**) a shared tumor-biology model, and (**c**) a drug-induced serotonin model

Our findings can be interpreted within three alternative explanatory frameworks: (1) a depression-related symptom burden model, (2) a shared tumor biology model, and (3) a drug-induced serotonin model as shown in Fig. 3.

First, consistent with our initial hypothesis, the depression-related symptom burden model proposes that lower patient-reported QoL may partly reflect depression or broader symptom burden, which could be associated with antidepressant use, lower tumor tissue serotonin abundance and poorer clinical outcomes. Low intratumoral serotonin levels together with prescriptions of antidepressants may be a marker for maladaptive (depressive) behaviors which may negatively affect survival in glioma patients. Such behaviors might include a tendency to opt out of treatments or to delay seeking care when needed. In addition, depression per se is associated with increased risk of early death related to self-destructive behaviors which may interact with the cancer diagnosis [29]. It should however be noted that depression was not directly measured in the present study, and QoL should not be interpreted as a proxy for depression. This model should therefore be regarded as a speculative explanatory framework rather than a directly tested mechanism.

Second, the shared tumor biology model proposes that low intratumoral serotonin levels may simultaneously induce oncogenesis and be a marker for depression. One possible interpretation is that the observed association arises from a direct connection between tumor biology and patient well-being. In this context, the plausible mechanism would be that a redirection of tryptophane metabolism through the kynurenine pathway, results in depletion of the substrates required for serotonin production. This hypothesis would be consistent with the fact that an increase in metabolites associated with the kynurenine pathway has been associated with a poor prognosis in cancer generally and in malignant gliomas in particular [30, 31]. If this interpretation would be correct, the clinical implication would be that treating depression, for instance with antidepressant medication, may improve QoL while not significantly affecting survival in glioma patients since treatment would work downstream of the oncogenic mechanism (i.e. affecting serotonin but not tryptophane and kynurenine levels). However, the fact that kynurenine and tryptophane metabolism were not associated with QoL or antidepressant use, and no clear associations were observed between these metabolites and survival makes this explanation less likely.

Third, a more speculative interpretation is the drug-induced serotonin mechanism, whereby antidepressant use may be associated with changes in intratumoral serotonin levels that are not fully captured in our cross-sectional measurements. That is, it remains possible that the metabolic data from the hospital-based cohort reflects an early stage prior to any potential increase in intratumoral serotonin following initiation of antidepressant therapy. In this scenario, antidepressant use might lead to higher intratumoral serotonin levels at later (postoperative) stages in the disease, which could theoretically exert oncogenic effects. Accordingly, if serotonin levels had been measured longitudinally, antidepressant use might have been associated with increased rather than decreased intratumoral serotonin levels. If such a mechanism would have been confirmed, one implication would be that pharmacological treatment with antidepressants could have negative effects on survival in glioma patients and should be avoided [13, 16, 17]. In sum, none of the proposed mechanisms can be definitively excluded based on the present study. Further research will therefore be needed to clarify their relative contributions.

### Limitations

Our use of two different datasets offering a combination of molecular, behavioral and epidemiological perspectives is an important strength of the present study. However, there are also several methodological limitations that need to be considered when interpreting our results. First, the cross-sectional design of both datasets precludes causal inference, meaning that all findings should be interpreted as associations rather than evidence of pharmacologic modulation. Second, the limited statistical power of our hospital-based cohort restricted our ability to explore several relevant issues in depth. For instance, there were too few individuals who both reported QoL and were treated with antidepressants to explore whether differences in QoL might account for the observed association between antidepressant use and intratumoral serotonin. As a result, the interpretation that the association between antidepressant use and low intratumoral serotonin may be explained by differences in QoL, although plausible, could not be directly evaluated. Moreover, information on adherence, dose, duration of preoperative antidepressant use was not available in the hospital-based cohort. Third, because QoL data was collected in a subset of patients and clinical management evolved over the study period, calendar-time effects and selection related to routine implementation of QoL assessments cannot be fully excluded. Forth, the limited power of the hospital-based cohort also means that the absence of associations between metabolites and survival should be interpreted with caution. Finally, the serotonin pathway metabolites were quantified in bulk tumor tissue obtained during surgery or biopsy and therefore represent metabolite abundance within the analyzed tissue specimen rather than direct measures of synaptic serotonin concentrations or central serotonergic neurotransmission. Consequently, the observed metabolite levels may be influenced by tissue heterogeneity, including variation in cellular composition, vascularity, inflammatory-cell infiltration, and other microenvironmental characteristics. In addition, information on factors such as tumor-cell content and blood contamination was not available. Therefore, the observed associations should be interpreted as relationships between tumor tissue metabolite abundance and clinical outcomes rather than as direct evidence of altered central neurotransmitter activity.

Regarding the population-based cohort, hypotheses concerning molecular mechanisms could not be directly tested. Furthermore, as this was an observational analysis, residual confounding cannot be excluded. Differences in patient characteristics, treatment patterns, functional status, or prescribing practices may have influenced both antidepressant selection and survival. Consequently, some degree of biological heterogeneity cannot be excluded, and the findings may not be directly generalizable to contemporary WHO 2021-defined IDH-wildtype glioblastoma. The present analysis focused on antidepressant exposure status and accounted for the timing of treatment initiation but did not model treatment discontinuation, switching between antidepressants, adherence, treatment duration, cumulative dose, or dose-response relationships. These factors may be relevant for both biological and clinical effects and warrant further investigation. Although time-varying modeling reduces the risk of immortal time bias, causal inference remains limited. Finally, it should be noted that fluoxetine and sertraline were analyzed as one group in the present study primarily because of a low number of patients treated with fluoxetine in the group. Conclusions regarding this compound may thus have to be taken with caution.

## Conclusions

From a clinical perspective, this study addresses the important question of whether antidepressants treatment influences outcome in patients with glioblastoma. The observed relationships between antidepressant use, tumor tissue serotonin levels, and patient-reported well-being suggest a complex interplay between serotonergic biology, clinical symptoms and antidepressant exposure. One plausible explanation is that these associations are driven by depression or symptom burden rather than direct oncogenic effects of antidepressants.

In the population-based cohort, survival differed across antidepressant exposure groups, although the observational design and incomplete availability of key prognostic variables preclude causal interpretation. The findings should therefore be considered hypothesis-generating rather than evidence of treatment effects. While the present results do not support the use of fluoxetine or sertraline as antitumor therapies, they provide no strong evidence that continued use of these medications is harmful when clinically indicated. Further studies are needed to clarify the underlying mechanisms and to disentangle the effects of antidepressant treatment from those of the conditions for which they are prescribed.

## Supplementary Information

Below is the link to the electronic supplementary material.

Supplementary Material 1

## Data Availability

The datasets generated during and analysed during the current study are available from the corresponding author on reasonable request.
