# Supplementary material for: Intratumoral serotonin and antidepressants in glioblastoma patients: narrowing the uncertainties
Source: J Neurooncol. 2026 Jun 30;178(3):84. doi: 10.1007/s11060-026-05694-1 (PMC13319255; doi:10.1007/s11060-026-05694-1)

**Supplementary**

**Figure S1**. Distribution of glioblastoma patients by operation year and availability of preoperative quality-of-life (QoL) data. Bars represent the number of patients operated each year, stratified by whether a QoL measurement was available (“with QoL”) or not (“without QoL”).


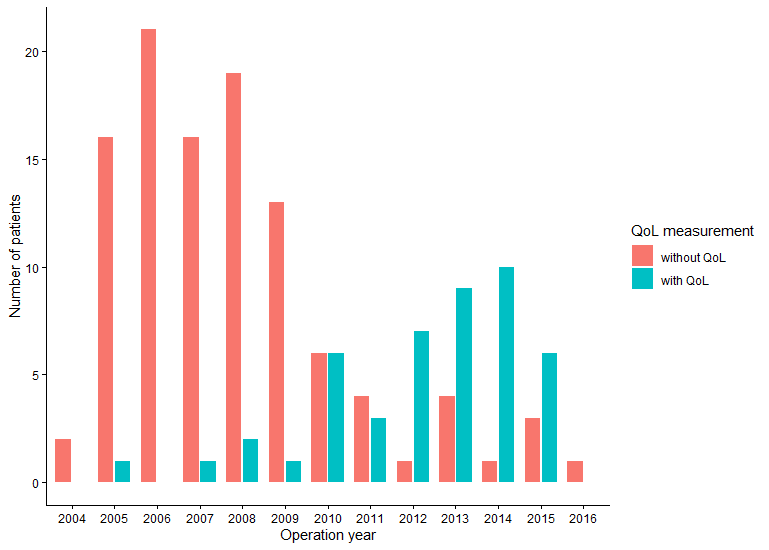

Supplement: Supplementary file 1 — Supplementary Material 1 [file 11060_2026_5694_MOESM1_ESM.docx]
